# Protocol for an online randomised controlled trial to evaluate the clinical and cost-effectiveness of a peer-supported self-management intervention for relatives of people with psychosis or bipolar disorder: Relatives Education And Coping Toolkit (REACT)

**DOI:** 10.1136/bmjopen-2017-016965

**Published:** 2017-07-18

**Authors:** Fiona Lobban, Heather Robinson, Duncan Appelbe, Johanna Barraclough, Emma Bedson, Lizzie Collinge, Susanna Dodd, Sue Flowers, Mahsa Honary, Sonia Johnson, Ceu Mateus, Barbara Mezes, Valerie Minns, Elizabeth Murray, Andrew Walker, Paula Williamson, Catherine Wintermeyer, Steven Jones

**Affiliations:** 1 Spectrum Centre for Mental Health Research, Division of Health Research, Lancaster University, Lancaster, UK; 2 Medical Research Council North West Hub for Trials Methodology Research, Department of Biostatistics, University of Liverpool, Liverpool, UK; 3 Division of Psychiatry, University College London, London, UK; 4 Division of Health Research, Lancaster University, Lancaster, UK; 5 eHealth Unit, Research Department of Primary Care and Population Health, University College London, London, UK

**Keywords:** health economics, relatives, mental health, digital

## Abstract

**Introduction:**

Despite clinical guidelines recommendations, many relatives of people with psychosis or bipolar disorder do not currently receive the support they need. Online information and support may offer a solution.

**Methods and analysis:**

This single-blind, parallel, online randomised controlled trial will determine clinical and cost-effectiveness of the Relatives Education And Coping Toolkit (REACT) (including an online resource directory (RD)), compared with RD only, for relatives of people with psychosis or bipolar disorder. Both groups continue to receive treatment as usual. Independent, web-based variable, block, individual randomisation will be used across 666 relatives. Primary outcome is distress at 24 weeks (measured by General Health Questionnaire; GHQ-28) compared between groups using analysis of covariance, adjusting for baseline score. Secondary clinical outcomes are carer well-being and support. Cost-effectiveness analysis will determine cost of a significant unit change (three-point reduction) in the GHQ-28. Costs include offering and supporting the intervention in the REACT arm, relevant healthcare care costs including health professional contacts, medications prescribed and time off (or ability to) work for the relative. Cost utility analysis will be calculated as the marginal cost of changes in quality-adjusted life years, based on EuroQol. We will explore relatives’ beliefs, perceived coping and amount of REACT toolkit use as possible outcome mediators. We have embedded two methodological substudies in the protocol to determine the relative effectiveness of a low-value (£10) versus higher value (£20) incentive, and an unconditional versus conditional incentive, on improving follow-up rates.

**Ethics and dissemination:**

The trial has ethical approval from Lancaster National Research Ethics Service (NRES)Committee (15/NW/0732) and is overseen by an independent Data Monitoring and Ethics Committee and Trial Steering Committee. Protocol version 1.5 was approved on 9 January 2017. All updates to protocols are uploaded to the National Institute for Health Research (NIHR) Journals Library. A full statistical analysis plan is available at https://figshare.com/account/home#/projects/19975. Publications will be in peer-reviewed journals (open access wherever possible). Requests for access to the data at the end of the study will be reviewed and granted where appropriate by the Trial Management Group.

**Trial registration number:**

ISRCTN72019945, pre-results.

**Strengths and limitations of this study**

- Assesses both clinical and cost-effectiveness of an intervention to increase access to National Institute for Health and Care Excellence-recommended support for relatives of people with psychosis or bipolar disorder.
- Entirely online trial design and embedded methodological studies to determine effective incentives for data collection.
- Peer-supported intervention with trained relative.

- No outcomes assessed for the people with psychosis or bipolar disorder being supported.
- Registration for the trial requires relatives to be currently distressed, which may exclude many relatives who still need this kind of support.

## Introduction

### Background and rationale

Relatives of people with psychosis/bipolar disorder (BD) provide a large amount of unpaid care,^1 2^ but at high personal cost in terms of distress and burden,^3–5^ and increased use of healthcare services.^6^ The UK Government recognises the need to support relatives in a caring role,^7^ and the National Institute for Health and Care Excellence (NICE) recommends all relatives are provided with information and support, and offered structured family intervention to enhance family coping and communication.^8 9^ However, a recent national audit of Early Intervention (EI) teams for psychosis showed poor implementation: only 50% of relatives are receivingreceiving a carer-focused education and support programme; only 31% offered structured family intervention and only 12% receiving it.^10^

Reasons for poor implementation are likely to be multifaceted but likely include limited time and resources within teams and among relatives. An online intervention to provide the necessary information and support to relatives may improve implementation. Online interventions are well established for many mental health conditions, including depression and anxiety,^11^ and are being rapidly developed for psychosis^12^ and BD.^13^ Such interventions are particularly suited to delivering standardised information and a platform to share ideas through online forums, but cannot replace structured family interventions. Online support is also being developed for relatives of people with other chronic health conditions,^14^ and may be particularly useful for these groups due to the flexibility of use, and empathy and support from being linked to other carers.^15^

We have developed the Relatives Education And Coping Toolkit (REACT) to provide high-quality information and support in an easy and free-to-access online form to relatives of people with psychosis or BD. If effective, it could help National Health Service (NHS) Trusts meet the national access and waiting times target for early intervention services to offer carer information and support to all.^16^

Online interventions may be best evaluated using online trial methodology to enhance the external validity of the trial.^17^ Advantages of online trial design include the potential to reach a greater number and range of participants more representative of the population likely to use an online intervention, to recruit more people over a shorter timeframe, to offer secure randomisation and data entry simpler protocols, and for a much cheaper trial due to fewer staff required.^18^ However, retention rates can be low,^18 19^ compromising internal validity of the trial. Offering incentives can improve retention rates,^20–23^ but there is considerable uncertainty as to the level of incentive required and whether the incentive should be conditional or unconditional on completion.

### Study aims and objectives

This randomised controlled trial aims to determine the clinical and cost-effectiveness of the REACT toolkit (which includes an online resource directory (RD)) and treatment as usual (TAU), compared with the RD only and TAU. This comparator was chosen to test the effect of offering REACT as an additional intervention to what relatives are currently able to access.

The objectives are to determine the following:

- impact of REACT on relatives’ distress
- impact of REACT on relatives’ well-being and support
- impact of REACT on hypothesised mediators of change, including relatives’ beliefs, perceived coping and amount of use of REACT
- costs associated with delivery and maintenance of REACT
- incremental cost-effectiveness ratio of REACT
- key issues for which relatives seek support.

We have embedded two methodological substudies. The objectives are to determine the effect on follow-up rates of the following:

- a low-value (£10) versus higher value (£20) incentive
- an unconditional versus conditional incentive.

The primary hypothesis is that there will be a significant difference (p<0.05) between the two arms of the trial in the General Health Questionnaire (GHQ-28) scores at 24-week follow-up.

## Methods

### Trial design

**Figure 1:**
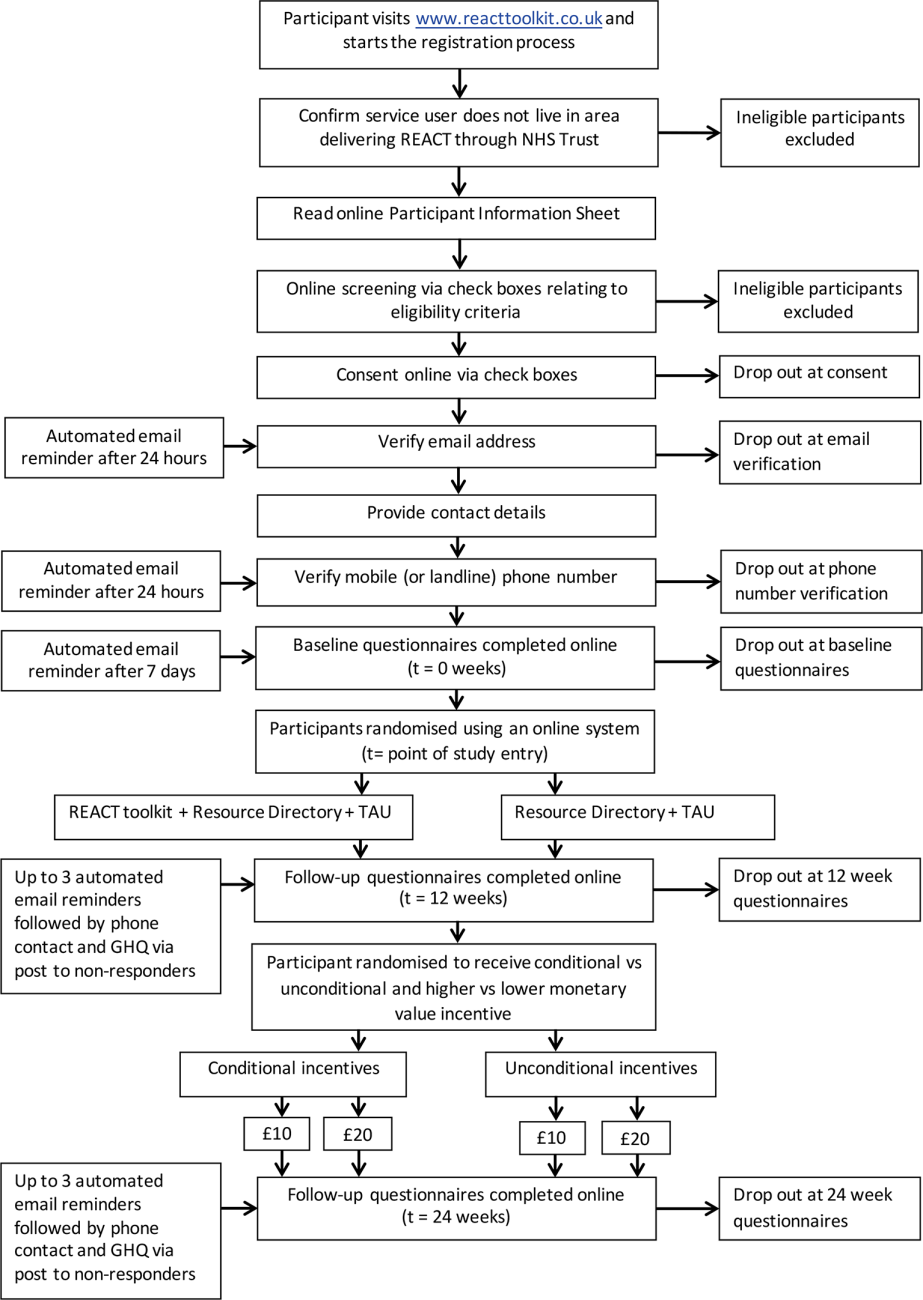
REACT trial flow chart showing participant pathway through the study. GHQ, General Health Questionnaire; REACT, Relatives Education And Coping Toolkit; TAU, treatment as usual.

This is a primarily online, two-arm, pragmatic, single-blind individually randomised controlled superiority trial. Participant pathway through the study is shown in figure 1.

### Public Patient Involvement (PPI) strategy

One of the study investigators and coauthor is a parent of someone living with psychosis and was extensively involved in the development of REACT, the RD and data collection processes. She is part of the supervisory team for the REACT supporters. We have a Relatives’ Advisory Group (RAG) working primarily online to provide detailed feedback on REACT toolkit, online data collection process and recruitment strategy. They will be involved in analysis, interpretation and dissemination of the data. Our Trial Steering Committee (TSC) includes people who are relatives supporting someone with a mental health problem.

### Setting: UK

This study will take place online in the UK. It is hosted by one NHS Foundation Trust, and other Trusts and clinical commissioning groups are eligible to take part as participant identification centres. Recruitment will also take place through local and national mental health charities, media, social media and Google Ads.

### Participants

#### Inclusion criteria

- Aged 16 or over
- Living in the UK
- Relative/close friend of someone with psychosis or BD
- Currently experiencing distress due to their relative or close friend (scoring ≥3 on the GHQ-28 item ‘Have you recently been feeling nervous and strung up all the time’)
- Currently help seeking (self-identified)
- Access to an internet-enabled computer
- Sufficient English fluency to comprehend intervention content
- Only one relative per service user may participate to avoid a clustering effect.

#### Exclusion criteria

As there is a parallel implementation study (called IMPART) of the same intervention (http://www.hra.nhs.uk/news/research-summaries/implementation-of-an-online-relatives-toolkit-impart-study), relatives living in any of the six areas where it is running will be excluded (by postcode).

### Recruitment

Recruitment is scheduled from April 2016 to October 2017. We will develop a social media strategy using Twitter, Facebook, Google Ads and blogging, and engage with local and national news media. We will recruit through national carer networks, MIND, Carers Trust, Rethink Mental Illness, Carers UK, SANE, Bipolar UK and NHS Choices. These organisations will all be listed in the RD in our trial. We will work closely with the Clinical Research Network to ensure information about the study is made widely available for recruitment through NHS Trusts and general practitioner (GP) practices. We will monitor the success of these strategies by asking all potential participants at registration (postconsent) to indicate how they heard about the trial and explore differences in demographics for those entering the study via different routes.

Based on previous studies our strategies to improve recruitment include a ‘lead-in’ period during which people can register interest in the trial during the set-up phase and be contacted directly when the trial starts,^24 25^ using online recruitment strategies to target those already using the internet to seek help,^19^ and paying participants.^20^

Potential participants will be directed to the study home page (www.reacttoolkit.co.uk), which provides study information. People visiting the site will provide the postcode of the person they care for to check they live in the UK, and not in one of the implementation study (IMPART) areas. Those in an IMPART area will be directed to the IMPART study site. Potential participants are directed to an online participant information sheet that details the study. Relatives are then asked to complete a short checklist to indicate whether they meet the inclusion criteria listed above.

Non-eligible relatives are invited to leave their contact details if they would like to be sent content of the REACT modules at the end of the study.

Eligible participants complete an online consent form and provide a valid email address. A copy of the consent form and a link to the registration process is sent by email (email validation step).

The registration process requires multiple contact details including mobile phone number, and checks for overlap with other participant details. A code is sent by text message to invite the relative to access the site. The data collection system requires email address and the registration name from the user. If any of the 7% of the UK population without a mobile phone take part, or there are any concerns identified through matching registration details, we will ask for a landline and verify identity using a code delivered by telephone. We will use postcodes to check all participants are living in the UK. We will request age and gender at baseline and also at 12-week and 24-week follow-up, as a final identity check. Similar strategies have been used successfully in previous trials.^23 26^

### Interventions

Participants can access the intervention site (REACT or RD) whenever they wish throughout the period of the trial (minimum of 24 weeks to last follow-up for final participant). They are advised to use the intervention according to the level of need. No changes are made to current treatment.

#### Development of REACT

REACT was developed with extensive input from clinicians and relatives through focus groups^27^ and the RAG. The content was first made available in paper/online pdf form, and feasibility and effectiveness in reducing relatives’ distress were demonstrated in a randomised controlled trial.^28^ Based on further qualitative feedback from participants in the feasibility trial, focus groups with relatives exploring their views of online interventions and RAG input, the content has been updated and expanded to offer a comprehensive online recovery focused toolkit for relatives of people with psychosis and/or BD. It includes online support from trained relatives (REACT supporters) via confidential direct messaging, and from other relatives through a restricted access forum moderated by the REACT supporters. Support offered by peers with a shared lived mental health experience is highly valued and can be as effective as support from health professionals,^29 30^ but there is no evidence on the effectiveness of relatives’ peer support. A key advantage of this design is that relatives of people who refuse to engage with services can be supported.

Each of the 12 key modules contains high-quality standardised written information, videos of clinical experts or experts by experience sharing their knowledge and experiences to illustrate key points, and self-reflection tasks to ensure content is personalised to the user. All videos of relatives telling their real story were retold by actors to preserve anonymity of those involved. A summary of each module is given in table 1.

**Table 1:** REACT information modules

Title	Description
What is psychosis?	Information about psychosis, what it feels like, possible causes and common misconceptions.
What is bipolar disorder?	An overview of bipolar disorder, its main features, the different presentations of bipolar and how it feels to experience it.
Managing ‘positive’ symptoms	An explanation of what is meant by the term ‘positive symptoms’, how these may be experienced, how they might appear to relatives and friends and how they can be managed.
Managing ‘negative’ symptoms	A detailed description of signs that make up ‘negative symptoms’, how these can manifest in people and how relatives can spot them. It explores how these might make relatives feel and gives suggestions as to how to help the person experiencing these symptoms.
Managing mood swings	Information on how to help people avoid extreme lows and highs, maintain a stable mood and support a relapse prevention/staying well plan. Suggestions are made on how to create a low-stress environment in a friendly, non-judgemental way.
Dealing with difficult situations	Describes the difficult situations that relatives and friends sometimes encounter, including risky, illegal or embarrassing behaviour, and offers ideas on how to manage these.
Managing stress — doing things differently	Helps relatives consider what stresses they have in their lives and how to adapt their own behaviours to help manage stressful situations.
Managing stress — thinking differently	Helps relatives consider how they are thinking about the stresses in their lives, and whether there are different perspectives that may help reduce their distress. Explores the many common thinking traps people fall into, such as ‘jumping to conclusions’ or ‘mind reading’, and helps relatives to test their own thoughts.
Understanding mental health services	Supports relative through understanding the complexities of mental health service provision to ensure they talk to the right people, to get the right information to meet their needs.
Treatment options	Information on what people with psychosis, bipolar disorder and also their relatives should be offered according to the NICE guidelines, including medication, psychological interventions and other therapies. Aims to empower relatives through outlining choices available.
Dealing with crisis	Clear suggestions about what to do in a crisis, where to turn to for help and what a relative should expect from services. Creates a very useful personalised ‘what to do in a crisis’ plan.
The future and recovery	Focuses on supporting recovery, with useful tips on how relatives can help people with bipolar disorder/psychosis develop confidence and gain independence, including finding a balance between support and enabling independence, looking for positive changes to celebrate, accepting new goals and challenges, and focusing on the bigger picture.

NICE, National Institute for Health and Care Excellence; REACT, Relatives Education And Coping Toolkit.

**Figure 2:**
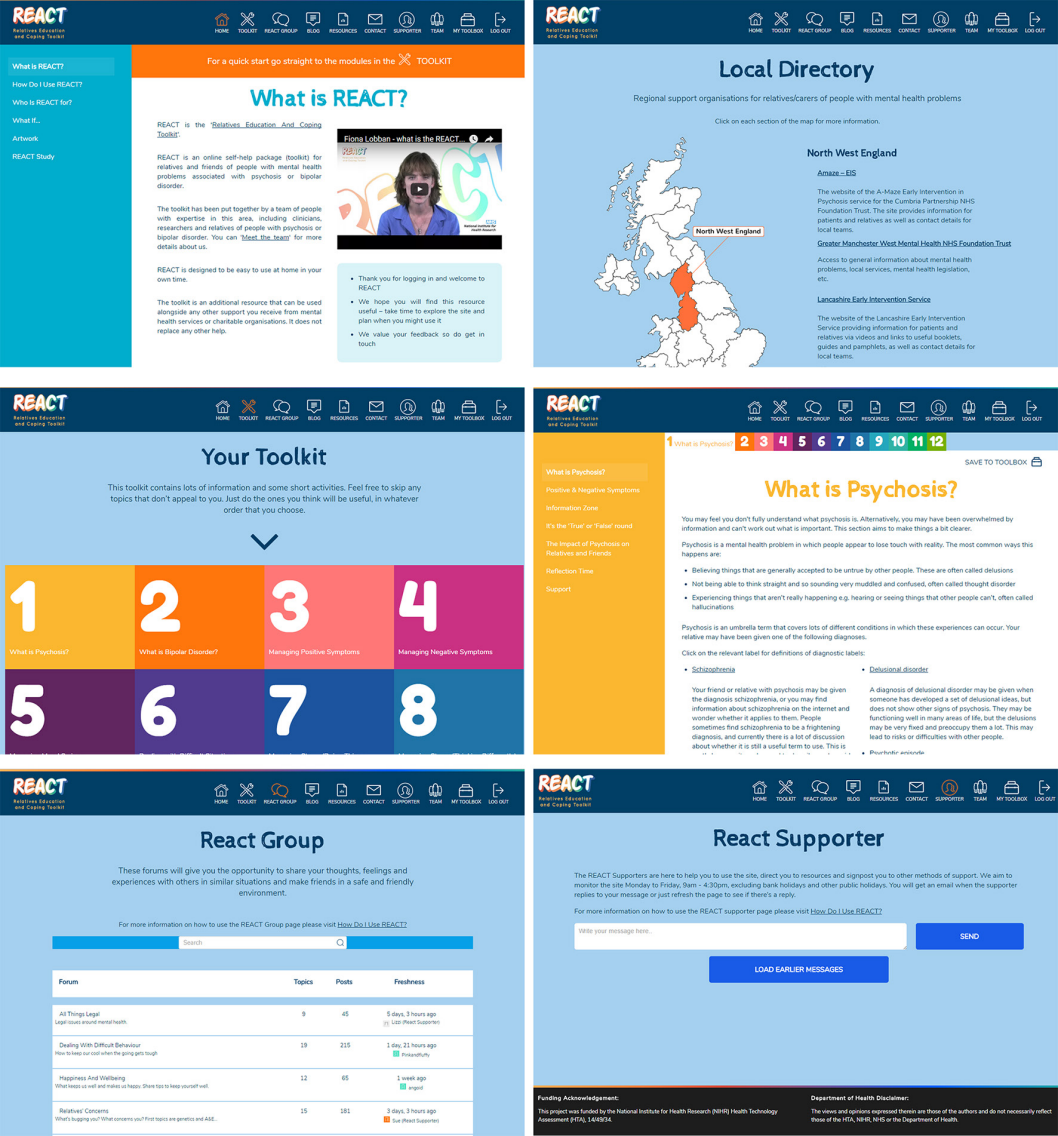
Screenshots to show look and feel of the Relatives Education And Coping Toolkit website.

A ‘Meet The Team’ page ensures relatives are fully informed about who is delivering the content of the site. Logos for Lancaster University, Lancashire Care NHS Trust, University College London, Liverpool Clinical Trials Research Centre (CTRC) and the McPin Foundation are prominently displayed on the login page. Mytoolbox offers user a confidential space to save links to any information sources they may want to access easily in future, including specific content within the toolkit, their self-reflection tasks and external web links. A blog page offers a flexible space for additional communication with site users, which can be edited by the REACT supporters.

Support is offered through confidential direct messaging with trained relatives (REACT supporters) and peer support through a moderated online forum.

Currently available national and local resources are listed in the RD. Screenshots in Figure 2 show the look and feel of the REACT intervention.

The REACT supporters are available on the site Monday to Friday, 09:00–16:30, excluding bank holidays and university holiday closures. Their key role is to provide emotional support and to guide relatives to relevant parts of toolkit and/or other relevant resources as appropriate. They are also trained to moderate the forum and can hide posts or withdraw access in response to inappropriate use. They are trained and supervised by a clinical psychologist and an expert relative.

Participants receiving REACT are sent reminders to visit the website after a week of inactivity. Participants can change the frequency of these reminder emails or turn them off.

#### RD arm

Participants allocated to RD arm log into the same website, but are only able to see the ‘Meet The Team’ and RD pages. At the end of the study those in RD only will be given access to the modules, without forum or direct messaging.

### Outcomes

**Figure 3:**
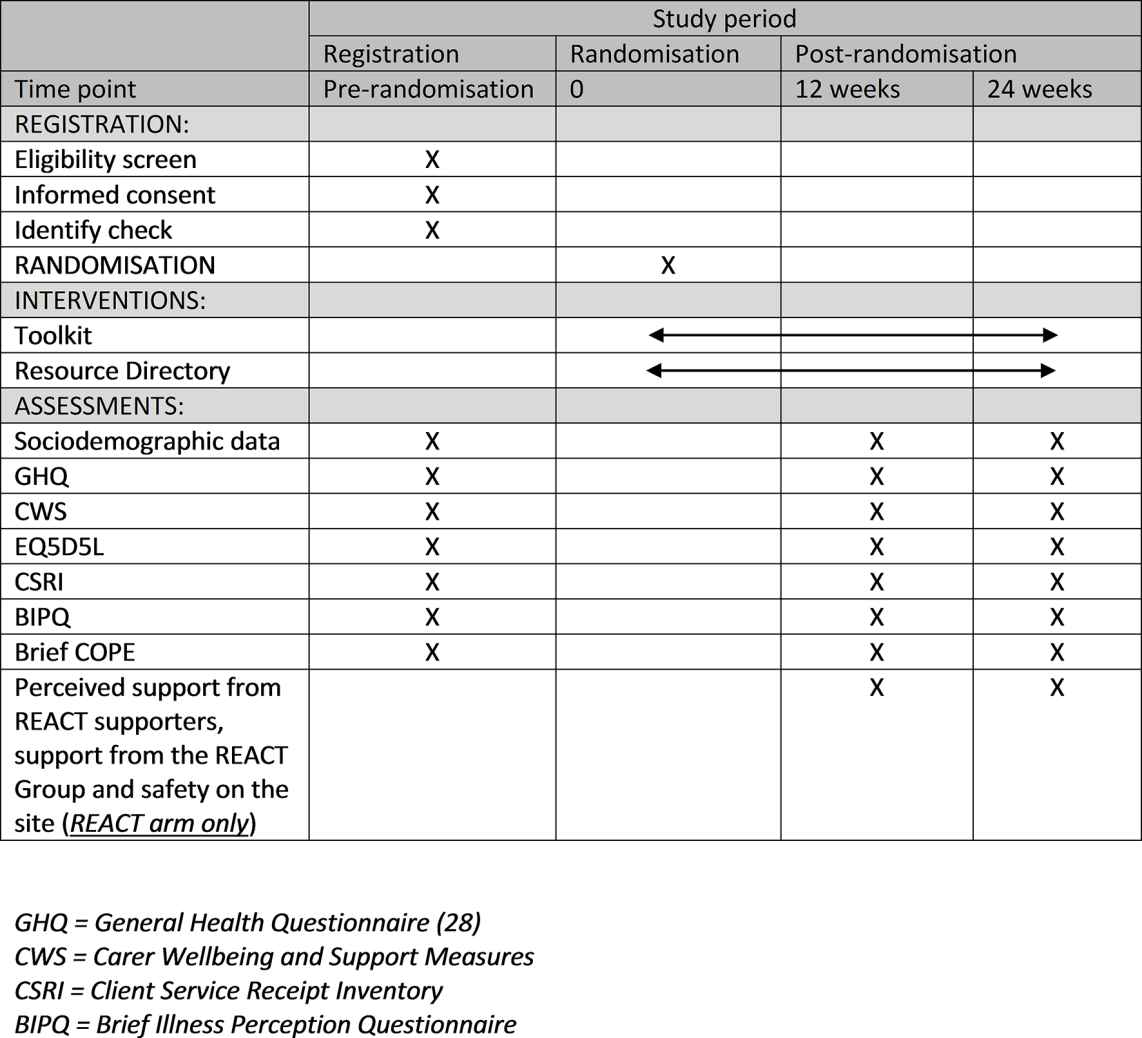
Measures and timing of completion.

Figure 3 shows all of the measures and when they are completed.

#### Primary outcome

The primary outcome is relatives’ distress at 24 weeks assessed using GHQ-28 with Likert scoring.^1–4 31^ GHQ-28 showed sensitivity to change previously^26 28^ and has shown significant associations with important functional outcomes in the general population, including GP visits,^32^ absence from work,^33^ incapacity benefits^34^ and severe adverse health outcomes, including deaths.^35^

#### Secondary outcomes

Secondary outcomes include the relatives’ experience of caring assessed using the Carers’ Well-Being and Support (CWS) measure^36^ assessed at 24 weeks, and distress (GHQ-28) and carer experience (CWS) at 12 weeks’ follow-up. CWS covers all aspects of carer’s experience of caring for someone with a serious mental health problem, including relationships, roles, financial concerns, physical/emotional health, stigma, worries about safety, satisfaction with support offered and ease of obtaining information.

#### Cost-effectiveness

We will determine the cost of a significant unit change (defined as three-point reduction) in GHQ-28. Cost utility analysis with fully incremental analysis will be calculated as the marginal cost of any changes in quality-adjusted life years (QALYs), using EuroQol EQ-5D-5L,^37^ as recommended by NICE.^38^ The EQ-5D-5L comprises five items covering the domains of mobility, self-care, usual activity, pain/discomfort and anxiety/depression.

Costs will include the direct costs of offering and supporting the intervention in the REACT arm, relevant healthcare care costs including health professional contacts, medications prescribed and time off (or ability to) work for the relative in both arms. An adapted version of the Client Service Receipt Inventory (CSRI)^39^ will be used to collect online retrospective information about the participant’s use of health and social care services, accommodation and living situations, income, employment and benefits in the preceding 6 months. We will include use of other free interventions including relatives support groups and websites, so we can accurately describe current treatment. Unpaid informal care by the relatives will be measured by asking relatives how many hours of care they provide supporting the person with mental health problems, and costing these on an hourly basis based on national mean age and gender-specific wage rates available from the Office for National Statistics.^40^ Days lost by relatives from work and reduced hours while at work due to the caring role will also be recorded and costed as part of the CSRI. Wherever possible, unit costs for medication and healthcare resources will be taken from national sources such as the British National Formulary^41^ and the Personal Social Services Research Unit (PSSRU) Costs of Health and Social Care.^42^

### Mediators of change

To test the proposed mediators of change in relatives’ outcomes, we will also include Brief Illness Perception Questionnaire,^43^ a 15-item Likert scale assessing beliefs about psychosis/BD with an additional single item, to assess perceived coping; and Brief COPE,^44^ a 28-item measure widely used to assess coping styles.

We will record all activity on the toolkit for each individual to test the relationship between use and effectiveness. Application Programming Interface data from the REACT site will be summarised for participants randomised to both intervention groups into a small number of variables (number of webpage downloads and time spent on the site).

In addition to amount of use, we will explore the nature of use, including qualitative analysis of forum posts and direct messages. We will also explore potential determinants of web use by assessing relatives’ experiences of the intervention for the REACT group only at 12-week and 24-week follow-up.

### Data collection

Baseline measures including demographic information, outcomes and mediators are completed before randomisation. Demographic data collected include age, gender, ethnicity, marital status, education, employment, living arrangements (including dependents), primary diagnosis of service user, length of time in caring role, number of people caring for, relationship to person(s) with mental health problem, whether or not they live with the person(s), level and type of contact, whether or not they are receiving support from NHS services and internet access.

Twelve and 24 weeks after randomisation, all participants are sent an email reminder to complete the follow-up measures. At 24 weeks, the email content will vary depending on whether participants have been randomised to either £10 or £20 reward and to the reward being conditional (ie, dependent on completion of follow-up questionnaires) or unconditional (ie, offered with the initial request for follow-up data).

To maximise follow-up at the primary outcome point we will:

1. only randomise participants once baseline assessment measures are completed
2. include detailed explanations in our recruitment materials to explain to participants why data completion at follow-up is so important
3. require email, telephone and postal contact details at registration so we have multiple methods of contact for follow-up
4. send participants up to three automated email reminders at 5-day intervals, followed by text, telephone and/or postal requests
5. incentivise completion of follow-up measures by paying participants shopping voucher(s) at each time point.

To increase overall acceptability and participation rates, we will inform RD participants that they will be able to access toolkit modules after the final follow-up.

### Sample size

We aim to recruit 666 relatives of people with psychosis/BD to accurately test the primary hypothesis that there will be a significant difference (p<0.05) between the trial arms in GHQ-28 at 24-week follow-up. Our feasibility trial^28^ showed a mean difference in GHQ scores between groups at 6 months (controlling for baseline) of 6.59 units (SD 16.6 units) in favour of the REACT arm. To build a degree of protection against pilot results proving optimistic, and to accommodate adaptations to the design of the study and the intervention, we reduce our estimate of the mean difference in this trial from 6.59 to 5.0 units. We retain our estimate of SD of 16.60 from the feasibility study, consistent with other studies using this measure with relatives in EI services^45^ and somewhat higher than those from other mental health or dementia services.^46 47^ Four hundred and sixty-six participants provides 90% power to reject the null hypothesis (p<0.05), with effect size of 5.0 units assuming 30% dropout by 24 weeks. Although dropout was only 17% in our feasibility trial, it is historically higher in online trials.^19^

### Internal pilot

Our trial includes a 9-month internal pilot with the following criteria:

- GO: 100% or above of anticipated recruitment at 9 months (333+ participants)
- AMEND: 80%–100% of anticipated recruitment (267–333 participants); review and amend recruitment strategies
- STOP: <80% of target for 9 months (<267 relatives); inform funders who will determine whether to stop trial.

If the SD of GHQ-28 scores at 24-week follow-up at the end of the internal pilot is higher than the estimated 16.6 units, the sample size will be recalculated and recruitment targets increased accordingly. If SD is lower, sample size will remain unchanged. If GHQ-28 retention at 24 weeks is less than 70%, recruitment target will increase to ensure 466 patients will provide sufficient 24-week primary outcome data to test the primary hypothesis.

### Randomisation

Eligible participants will be randomised using a 1:1 ratio to ‘REACT (including RD)+TAU’ versus ‘RD+TAU’ by the CTRC. We will use web-based variable block randomisation in which the unit of randomisation is the relative. We will explore the effect of sociodemographic and caring-related variables; however, without convincing evidence these will have an effect, we have not stratified randomisation.

A second independent randomisation is carried out at CTRC at 24-week follow-up using a randomised factorial design using the same individual block randomisation, with participants randomised to £10 or £20 reward (shopping voucher) and to the reward being conditional or unconditional on completion of measures to determine relative effectiveness and costs for each reward strategy.

### Allocation concealment and blinding

All data are self-reports and predominantly input online by participants. Where data are collected by post, these will be recorded and inputted by the trial manager blind to allocation. Data are uploaded directly to an electronic database at the CTRC. The system only allows valid values to be entered. To prevent any bias in the conduct of the study, the chief investigator (FL), trial manager (HR) and statistician (SD and PW) will be blinded to treatment assignment. Participants, REACT supporters (LC, SF, CW), clinical supervisors (SoJ, StJ) and technical staff are unblinded.

To minimise unblinding any contact with participants will be prefaced by a reminder not to disclose trial arm. If the trial manager is unblinded, then non-automated reminders and any data entry will be done by another blind team member. Chief Investigator (FL) will be unblinded only in the case of a serious adverse event deemed to be study-related to ensure the event is appropriately reported and investigated. All instances of unblinding will be recorded.

### Data management storage and security

All participant trial data are collected through an online system at CTRC and stored on secure servers physically located within access-controlled server rooms and backed up nightly to a separate physical location. All identifiable data are encrypted using a 256-bit encryption algorithm. CTRC servers are subject to penetration testing audits undertaken by the University of Liverpool central IT staff. Website usage data and qualitative data from the REACT group and REACT supporter direct messages are taken from the REACT toolkit hosted on a dedicated virtual private server at Lancaster University. All communication with website users is limited to SSL-protected HTTPS protocol to protect passwords and data in transit over internet.

## Data analysis

A full statistical analysis plan is available at https://figshare.com/account/home#/projects/19975. If normally distributed, scores on the primary and secondary outcomes will be summarised using means and SDs for each arm separately, and will be compared between groups using analysis of covariance, adjusting for baseline score, and including all participations according to the randomisation scheme. If the scores are not normally distributed, the median and IQR will be presented for each randomised group and will be compared using the Mann-Whitney U test. An appropriate transformation (eg, log) will be applied, and analysis of covariance will be applied to data, adjusting for baseline score.

To investigate the relationship between website use and outcome, data will be recorded on baseline covariates (correlated with both website use and outcome) and relevant website use (from participants in both arms). Instrumental variable regression will be implemented to estimate impact of website use on the primary outcome (GHQ-28 at 24 weeks), as well as to test whether the mediator variables actually predict change in outcome. Mediating variables will be examined individually in this exploratory analysis.

To assess the impact of the second randomisation, the number (proportion) of participants providing 24-week follow-up data will be presented and compared using the χ^2^ test (or Fisher’s exact test, if expected counts are <5). The independent impact of intervention group on retention rates will be explored by including intervention group along with value of the reward (or un/conditional nature of the reward) as an explanatory variable in logistic regression.

### Cost-effectiveness

Cost utility with a fully incremental analysis using an NHS perspective at 24 weeks will be done. Effectiveness will be assessed by changes on GHQ-28. EQ-5D-5L will be used to generate QALYs. Uncertainty around cost-effectiveness estimates will be explored using cost-effectiveness planes (through generating a large number of cost–outcome combinations using bootstrapping) and cost-effectiveness acceptability curves (showing the probability of the intervention being cost-effective at various levels of willingness to pay). This allows any uncertainty in the costs or outcomes to be reflected in the results presented. The NICE Health Technology Assessment (HTA) guidance will be followed. However, costs of informal support can impact on cost-effectiveness when it constitutes a substantial part of the support provided, so we will account for this by also providing results from the wider societal perspective including estimates of carers’ productivity losses.

### Missing data analyses

To minimise missing data, participants are required to complete the primary outcome measure (GHQ-28) before completing any other measures. Participants are unable to submit any questionnaire with missing fields, thus avoiding missing data within questionnaires. As much data as possible will be collected about the reasons for missing data, and these will be used to inform the handling of missing data. Participants will be invited to give reasons for not responding to the email reminders.

The baseline characteristics of those who do/do not provide data will be compared to demonstrate whether missing data can be assumed to be missing at random (at least with respect to recorded baseline characteristics). A joint modelling approach (using baseline, 12-week and 24-week outcome data) will be used to assess the impact of missing data at 24 weeks on the conclusions drawn from analysis on primary and secondary efficacy outcomes.

Participants are free to withdraw consent from the trial at any time without providing a reason, although we invite them to tell us why they have withdrawn so that we can take this into consideration in future studies.

## Monitoring

The trial is overseen by an independent Data Monitoring and Ethics Committee (DMEC) including Professor of Trials and Professor of Clinical Psychology, and the TSC Chaired by Professor of Clinical Psychology and including a trials statistician, trial methodologist and expert relative, both funder-appointed (National Institute for Health Research (NIHR)). The TSC will oversee trial progress, ensure that it is being carried out according to protocol and decide on continuation at the end of the internal pilot. DMEC will review unblinded data and prioritise participant safety, alerting TSC to any concerns regarding safety or other ethical issues. TSC will liaise directly with the trial sponsors (Lancaster University) who may audit the trial at any time.

The number (and percentage) of patients with at least one major/minor protocol deviation will be summarised by treatment group. Eligibility protocol violations and multiple registrations per participant or per service user will also be reported.

### Adverse events

Adverse events are defined as either low risk (clear evidence of distress or concerns of risk of harm or abuse towards participants or others, but no immediate or serious threat of severe harm or risk to life) or high risk (clear evidence of immediate risk to life or child welfare). Risk can be identified through online questionnaire red flag items, posts on the REACT group, direct messages to REACT supporters and by the trial manager during email or telephone participant contact. Low-risk events will be discussed in supervision, documented and trigger a standardised email expressing concern and providing details of how to seek crisis support. If an immediate high risk is identified, either the police (immediate risk to life) or social services (risk to child) will be contacted as appropriate. Risk will be reported to the supervising clinician and documented. The supervising clinician will discuss the risk event with the TSC Chair, who will decide if the event is related or unrelated to the study. If related, Chief Investigator and Trial Manager will be unblinded, and the sponsor, ethics committee and funding body will be notified. The number of adverse events and how they were identified will be recorded for both arms of the trial.

## Reporting and dissemination

The trial will be reported following the Consolidated Standards of Reporting Trials guideline.^48^ The International Committee of Medical Journal Editors guidelines on authorship will be followed. Products will be widely disseminated through journal articles, conference presentations and social media to all relevant stakeholders internationally, including service users, relatives, NHS managers and frontline clinical staff including GPs, clinical academics and the general public. A study website will provide updates and outputs from the study and links to all publications and presentations. Data will be stored at Lancaster University and the Trial Management Group, which consider applications for access to the data for further analyses.

## Financial arrangements

This trial is funded by the NIHR, HTA, 14/49/34. Contractual agreements are in place between the sponsor (Lancaster University), the CTRC (Liverpool University) and University College London, and Lancashire Care NHS Foundation Trust, which incorporates financial arrangements. The REACT trial is supported by the Comprehensive Local Research Network including research support costs. Participants are covered by indemnity for negligent harm through the standard NHS indemnity arrangements. Lancaster University has insurance to cover for non-negligent harm associated with the protocol.

## Supplementary Material

Reviewer comments

Author's manuscript
