# Supplementary material for: Protocol for an online randomised controlled trial to evaluate the clinical and cost-effectiveness of a peer-supported self-management intervention for relatives of people with psychosis or bipolar disorder: Relatives Education And Coping Toolkit (REACT)
Source: BMJ Open. 2017 Jul 18;7(7):e016965. doi: 10.1136/bmjopen-2017-016965 (PMC5541455; doi:10.1136/bmjopen-2017-016965)
Supplement: Author's manuscript [file bmjopen-2017-016965.draft_revisions.pdf]

PEER REVIEW HISTORY

BMJ Open publishes all reviews undertaken for accepted manuscripts. Reviewers are asked to complete a checklist review form (**http://bmjopen.bmj.com/site/about/resources/checklist.pdf**) and are provided with free text boxes to elaborate on their assessment. These free text comments are reproduced below.

ARTICLE DETAILS

**TITLE (PROVISIONAL)**	Protocol for an online randomised controlled trial to evaluate the clinical and cost effectiveness of a peer supported self-management intervention for relatives of people with psychosis or bipolar disorder: the Relatives Education And Coping Toolkit (REACT).
**AUTHORS**	Lobban, Fiona; Robinson, Heather; Appelbe, Duncan; Barraclough, Johanna; Bedson, Emma; Collinge, Elizabeth; Flowers, Susan; Honary, Mahsa; Johnson, Sonia; Mateus, Ceu; Mezes, Barbara; Minns, Valerie; Murray, Elizabeth; Walker, Andrew; Williamson, Paula; Wintermeyer, Catherine; Jones, Steven

This article was not externally reviewed at BMJ Open. Protocols that have been independently assessed prior to submission to BMJ Open are usually fast-tracked to publication on the grounds that further substantial changes will not be possible. This independent assessment will usually be external, independent review for both a substantial grant award from a non-commercial or government funder and ethics approval.
